# Are School Feeding Programs Prepared to Be Inclusive of Children with Disabilities?

**DOI:** 10.3389/fpubh.2016.00045

**Published:** 2016-03-17

**Authors:** Sergio Meresman, Lesley Drake

**Affiliations:** ^1^Inter American Institute on Disability and Inclusive Development, Montevideo, Uruguay; ^2^The Partnership for Child Development, London, UK

**Keywords:** disabled children, school health services, nutritional status, equity, disability

## The Last 10%

According to the World Health Organization and the World Bank “approximately 93 million children in the world – 1 in 20 children aged 14 or younger – have a moderate or severe disability of some kind. The majority of them live in the undeveloped world, are not enrolled in school, and have very poor access to the most basic health and nutrition opportunities. In low- and middle-income countries, children with disabilities are more likely to be out of school than any other group of children” (1). Girls with disabilities are particularly disadvantaged and most likely to be excluded from education (2).

Due to poor data and a lack of knowledge about their needs, school health and nutrition programmers are challenged to respond effectively. However, lack of visibility is not the only barrier to children with disabilities receiving school health and nutrition. Structural challenges do exist for children and adults with disabilities to access health and education services, most noticeably, inadequate infrastructure (2).

The ratification of the International Convention on the Rights of Persons with Disabilities has had significant impact in most countries. It has brought up a number of new challenges for public policies to be inclusive of children with disabilities in existing and new programs. Such a challenge can be summarized in one insight: it is now clear that the development and sustainability goals of the world cannot be achieved without the full and effective inclusion of the most vulnerable children, many of whom are children with disabilities.

## Malnutrition and Disabilities

The intersection between nutrition and disability has been largely overlooked in both programming and research. This is a significant gap in knowledge and policy making, given that over one billion people are undernourished and more than one billion live with a disability (1).

Malnutrition is found worldwide and is directly or indirectly linked to major causes of death and disability. Deafness, for instance, is most often the result of malaria, typhoid, and meningitis, which are highly prevalent in many undeveloped regions of the world. Other diseases, such as foodborne or waterborne diarrhea, often result in a disability. In the developing world, malnourishment and disability severely limit life opportunities. Both are global development priorities as well as crucial equity and human rights issues. In spite of this, the two issues are rarely linked.

Although research and experience have largely proven the relationships among nutritional status, cognition, school participation, and academic achievement (3), the nutritional needs of children with disabilities are rarely considered in the design of school feeding programs. Inclusive School Nutrition programs are intended to significantly help children with disabilities by promoting their educational access, retention, and learning outcomes.

## Primary Barriers

Several barriers prevent children with disabilities from receiving nutrition outreach efforts. Some are structural: persons with disabilities face numerous challenges to access health and social services; little available data makes them invisible as a population group; and, consequently, most education and health initiatives are not disability inclusive. Regular health and education personnel are often unprepared to communicate effectively with children with disabilities, and health and nutrition education programs often fail to consider their learning needs (4).

Other barriers could be more “systemic” (related to the functional characteristics of programs and responsible institutions) and might take various forms:

- Dining halls and other important premises such as washrooms and toilets are often not accessible to those with mobility impairments.^1^
- Because of a lack of experience with persons with disabilities, some teachers as well as other school personnel and health staff are unaware of the codes of interaction that are needed to work effectively and comfortably with children with disabilities^2^ (4).
- Some children might require assistance in feeding. School personnel responsible for preparing meals may not have the skills or awareness of the specific needs of children with disabilities in terms of hygiene, eating, or swallowing.
- Special dietary restrictions might be required.
- Even when nutrition topics are systematically included in school curricula and health topics associated with nutrition are included in cross-curricular activities, curricular adaptations, or teaching resources to make these contents accessible and affable to children with disabilities are barely accessible.
- Discrimination is often present due to stigma. For instance, children without disabilities could be given priority by school personnel based on the belief that their safety and well-being are of greater value than those of children with disabilities.
- Some children might be ostracized and face negative attitudes in the same way they face physical barriers. Fostering inclusion will always require overcoming these obstacles and confronting long-standing taboos created by prejudice.

## What is “Inclusive”?

Over the years, the term “inclusive education” has been used to refer to “including children with disabilities,” for example, children who have visual impairments, hearing limitations, reduced mobility, or experience difficulties learning in “regular” classrooms. Inclusive education is an important strategy for the most vulnerable groups, for example, “children who don’t speak the common classroom language or belong to a different religion or caste, and children who may be at risk of dropping out because they are sick, hungry, or not excelling academically” (3). The term can also include girls who are pregnant, children affected by HIV/AIDS, and all girls and boys who are not enrolled in school, especially children who are employed in order to help their families survive.

Within the Focusing Resources on Effective School Health (FRESH) framework, inclusion means having proactive policies and plans to ensure learning for all children, particularly those who are at risk of being left out or excluded from school (5). The question of whether school feeding programs and services are prepared to be inclusive of children with disabilities mandates that all these children will be included and accounted for.

## Delivering School Feeding to Children with Disabilities

There are two main ways of delivering school feeding programs to children with disabilities:

- Through regular school feeding in mainstreaming education.
- Including special education institutions in school feeding.

There is often disagreement over whether children with disabilities are more effectively reached through “special education” or regular, universal programs. The alternative approach is to make educational resources more accessible to all persons, with and without disabilities. While a significant percentage of children remain in specialized institutions, growing consensus indicates that including children with disabilities in the mainstreaming sector is both cost effective and socially effective and leads to overall improvements in educational outcomes. However, in most cases, this debate is futile because both strategies are valid and necessary. Furthermore, they can easily be combined. The combination of “mainstreaming” disability in all nutrition projects and having disability-specific interventions available is called the “twin-track” approach (4).

## Principles for Inclusion

Implementing inclusive school feeding programs is not necessarily complex. As described in Partnership for Child Development, three principles were identified that need to be addressed (4).

1. Include children with disabilities *in the planning process*:
  Improved consultation will help to make this group visible and increase the likelihood that programs are sensitive to their needs.
2. Ensure that *accessibility* is guaranteed throughout the delivery plan:
  Addressing issues of physical access to the places where feeding services are delivered and providing nutritional information and education in a way that is accommodated to the children’s needs and functional styles is essential:
  - Accessibility to the physical environment: the school compound, dining hall, wash rooms, and toilets (see standards on physical accessibility).
  - Accessibility to nutrition information and education: Providing access to nutrition information and communication is crucial for children with disabilities to decrease their risk of infections and improve their health and nutrition-related decisions. Materials and campaigns, including printed materials, media-based campaigns, and digital contents offered over the internet, must be designed to effectively reach children with different types of disabilities and take into account their diverse functional characteristics (4).
  - Training: pre- and in-service training is needed to improve the knowledge of staff to appropriately respond to the needs of children with disabilities.
3. Promote family and community *awareness* and *participation*:
  Engaging families and community organizations such as local disability organizations, churches, special education centers, and rehabilitation programs is of great support to the effective and sustainable implementation of inclusive school feeding programs. Such an approach will have mutual benefits for school programs and school health and nutrition teams, as well as parent organizations, which often have little information and awareness about school feeding programs and other available resources.

## Special Dietary Needs

The recommended course of action in situations involving substitutions for special dietary needs is to encourage school personnel to work closely with families and provide supervision of the appropriate professionals to ensure that schools make *reasonable* accommodations to allow for the child’s participation in the meal service (6). In most cases, special dietary needs can be met reasonably through a diet order that meets the program standards and is appropriate for the child.

## Check List

The following check list is proposed to conduct a rapid assessment of the conditions for participation of children with disabilities at school level. It seeks to ensure that all key components for inclusion are in place.

### Accessibility

- Is the school compound accessible?
- Is there accessible transportation to come to the school?
- Are school meals accessible? Have adaptations been made to respond to needs of children with disabilities?
- Are health activities accessible? Have adaptations been made to respond to needs of children with disabilities?
- Is the drinking water accessible to all children?
- Are latrines/toilets and handwashing facilities accessible to all children?
- Is the playground accessible to all children?

### Attendance

- Are there children with disabilities in school?
- Have there been children with disabilities in the recent years that have dropped out?
- Have schools located and contacted all children with disabilities in the area?

### Inclusion

- Number of teachers trained on inclusion.
- Number of teachers with experience on inclusion.
- Adaptations to the curriculum.
- Adaptations to assessments/tests.
- Assistive devices and teaching resources for blind/low vision.
- Assistive devices and teaching resources for those with hearing impairments.
- Teaching resources for intellectual disabilities.
- Teaching assistants available.

### External Alliances, Partnerships, Support

- Support from itinerant teachers and local inclusive education resource centers
- Support from disability organizations
- Support from parents and parents’ organizations
- Participation of parents of children with disabilities in parents’ organizations

## Conclusion

School feeding programs urgently need to go through inclusiveness assessments as well as engage in consultation with families and disability organizations to ensure that their rights are fulfilled and their participation contributes to closing the equity gap. This is essential as children and adolescents with disabilities constitute a significant proportion of the population, especially in the most deprived regions of the world (3). The goal of development and education for all must incorporate the full and effective inclusion of the most vulnerable children, many of whom are children with disabilities.

## Author Contributions

Prof. SM has been working in community and school-based health and inclusion for many years. In this article, his expertise benefited from the extensive experience brought over by Dr. LD who directs the Partnership for Child Development and is a worldwide expert on school nutrition. Dr. LD and her PCD team provided the programmatic framework and the financial support that allowed Prof. SM to explore the inclusion of children with disabilities in existing school feeding programs. Prof. SM led the process of consulting several specialists on the ground and proposed the question around which the topic is explored. The result is this opinion article that proposed a platform for future research.

## Conflict of Interest Statement

The authors declare that the research was conducted in the absence of any commercial or financial relationships that could be construed as a potential conflict of interest.

## Footnotes

^1^“From 78 schools in Kenya, only in 5 I have seen accessible toilets” (Personal communication, Department of Disability Mainstreaming, Ministry of Labor and Social Affairs, South Africa)

^2^“Quite often, feeling that you are unable to help a child with a disability or been just intimidated by a potential difficulty, results in children been sent back to their homes at the school lunch time” (Personal communication, School Feeding staff at the Partnership for Child Development Office in Kenya)
